# A Class of Pulpless Teeth, Their Treatment

**Published:** 1883-01

**Authors:** A. M. Ross

**Affiliations:** Chicopee, Mass.


### ARTICLE IV.

### *A Class of Pulpless Teeth, their Treatment.*

BY A. M. ROSS, CHICOPEE, MASS.

I have prepared this paper without reference to the literature of the subject of treating pulpless teeth, it being rather a result of long experience with a certain substance with the rationale of its application to a certain class of teeth. I know that the substance is used, to some extent, by dentists to accomplish the result for which I use it, but I do not recollect of ever reading any *scientific* reason *why* it is used. About eight years ago, while in practice with my father in Troy, N. Y., I had my attention called to the permanganate of potassa by a friend, a druggist in the city. We made many experiments with it—not confining our attention to spittoons—to prove its antiseptic property, and we were surprised at the time by the fact that but a small amount of the crystal was necessary to decompose a comparatively large amount of putrefactive matter.

Since that time I have used it extensively in my practice in treating a certain class of pulpless teeth.

I will not say that the subject of treating this class of teeth is an uninteresting one to dentists, but my methods and reasons for them may be quite uninteresting, and I will use as few words as possible in so doing.

I believe that the use of the permanganate of potassa in all teeth where decomposition of the pulp has occurred, is attended with greater certainty of result favorable to the pericementum and subjacent tissues than by the use of salicylic acid, carbolic acid, and other preparations from phenol. Of the derivations of phenol it may be safely asserted that they *prevent* decomposition of normal albuminous matter by coagulation, deeply or superficially —but that they do not, or cannot decompose products of albumen, their action being a coagulating one rather than a decomposing one, I refer to the contents of the walls of the pulp cavity and do not wish to be understood as applying my remarks to pericementum for this reason: Upon the death of the pulp the albuminous matter in the dentinal canaliculi coagulates at first, and thus the structure is rendered opaque. This is the first change that occurs, which is followed by the decomposition of the coagulum. Now if tho pulp is removed before death, and the cavity immediately saturated with carbolic acid, a coagulum is formed that is not so easily decomposed; or if the treatment is adopted after the removal of an arsenized pulp, the result may be as good, because arsenic does not coagulate albumen, if our esteemed friend, Prof. Mayr, is correct. (I would say in parenthesis that in view of the circulation that is maintained in the cementum and the periphery of dentine of " dead " teeth, and the fact of long continued action of arsenic, unless the arsenized fluids can be coagulated, I think that something else than arsenic for destroying pulps should be used.)

Death of the pulp is followed by decomposition. Decomposition, of the pulp from any physiological or pathological cause will produce the sulphides of albumen, sulphuretted hydrogen, etc.

After the pulp-chamber of the tooth has been freely opened, so that the canal or canals may be entered by a smooth broach, less than a grain of the potassa is placed in a half ounce of cold water, or about in that proportion, about a drachm being prepared for a treatment. A little absorbent cotton is roped on the broach, saturated in the solution and introduced but a short distance, and immediately removed. The color is changed. The fluid as introduced is a beautiful purple; upon removal it is brown. In this way a number of twists of cotton are wet in the solution, each in turn forced further into the canal, until the bits of cotton upon removal are found to be but little changed in color from purple. Thus I have used as many as twenty little twists of cotton in a *first* treatment. By taking precaution to never make the solution too strong, I have obtained most satisfactory permanent results. I have frequently conversed with Prof. Mayr upon this subject and have been enlightened upon it very much. I will briefly state that the permanganate of potassa is maganese peroxide, plus oxygen, plus potassium; that there is an excess of oxygen ready for combination; that this oxygen being brought into a cavity containing sulphides of albumen or sulphuretted hydrogen, these are decomposed and water is formed, etc., and the permanganic acid is thus reduced to the peroxide of manganese, or oxide of manganese, according to circumstances, both of which are brown in color. The use of this chemical in treating a certain class of pulpless teeth insures the decomposition of those products that are the feeders of pyogenic membrane, and the sense of *sight* being quite sufficient to make apparent the accomplishment of this. We hear much talk about the ease and certainty with which certain bicuspid and molar buccal roots are opened, cleansed and filled with gold, etc. A careful study of *many* such roots by transverse sections under the microscope would show how utterly impossible this is in a great majority of cases, and how impossible it is to conjecture about the size and character of canal by outside formation, and we don't even have this to guide us except in cases of replantation.

I speak of this to show how impracticable much of this talk is about *mechanical* dexterity in treating and filling pulp canals. It is misleading to those who do not know much of tooth anatomy. In many cases it is not possible to reach the apices of roots, and the closing up of that portion of the root *has to be done as near* the apex as possible. Now if the canals in many bicuspids are plural, elliptical in transverse shape, in buccal roots of superior molars the same, how are we to be certain of a good result in any case except where we use an agent that shall form new compounds and leave a veritable and comparatively insoluble new product in place of the old decomposing one? An agent that can be flooded over all surfaces? This agent *does* stain dentine, but so slightly if the proportions of crystal to water are right for each case that the surface of the tooth does not reveal it. But is not the oxide or peroxide of manganese preferable as a deposit in the open ends of the dentinal canaliculi than some of the products of decomposed albumen? The proper *treatment* of this class of pulpless teeth is of as much importance as the after *sealing of*

*ends of the canal;* both operations being thorough, the material with which the *root is filled* is of very little importance.—*New Eng. Journal of Dentistry.*

